# Contextual Determinants of Clinical Pharmacists’ Contributions to Team-Based Antimicrobial Stewardship in Jordanian Hospitals: A Realist-Informed Qualitative Study

**DOI:** 10.3390/antibiotics15070670

**Published:** 2026-07-08

**Authors:** Mona Bustami, Saba Ammar Alabdali, Mohammad Abu Assab, Inas Almazari, Wafa’ A. Al-Haj, Wael Abu Dayyih, Hayam A. Alrasheed, Zainab Zakaraya, Anas Abed

**Affiliations:** 1 Faculty of Pharmacy, University of Petra, Amman 11196, Jordan; mbustami@uop.edu.jo; 2 Department of Biopharmaceutics and Clinical Pharmacy, Faculty of Pharmacy, Al-Ahliyya Amman University, Amman 19328, Jordanz.zakaraya@ammanu.edu.jo (Z.Z.); 3 Clinical Pharmacy Department, Faculty of Pharmacy, Zarqa University, Zarqa 13132, Jordan; mabuassab@zu.edu.jo (M.A.A.); ialmazari@zu.edu.jo (I.A.); 4 Faculty of Pharmacy, Al-Ahliyya Amman University, Amman 19328, Jordan; 5 Faculty of Pharmacy, Mutah University, Alkarak 61710, Jordan; wabudayyih@mutah.edu.jo; 6 Department of Pharmacy Practice, College of Pharmacy, Princess Nourah Bint Abdulrahman University, P.O. Box 84428, Riyadh 11671, Saudi Arabia; haalrasheed@pnu.edu.sa

**Keywords:** clinical pharmacy, antimicrobial stewardship, antimicrobial prescribing, hospital pharmacists, team-based care, qualitative research, realist-informed analysis, interprofessional collaboration, Jordan

## Abstract

Background/Objectives: Clinical pharmacists contribute pharmacotherapy expertise to antimicrobial stewardship (AMS), but antimicrobial prescribing remains a multidisciplinary process led by treating clinicians. In physician-centered hospital systems, pharmacists’ contributions may be integrated into prescribing decisions to varying degrees depending on the organizational structures, interprofessional relationships, workflow integration, and prescribing cultures. This study aimed to explore how contextual and interprofessional factors shape the integration of clinical pharmacists’ antimicrobial stewardship contributions within multidisciplinary prescribing decisions in Jordanian hospitals. Methods: A multi-site qualitative study was conducted across nine Jordanian hospitals. Semi-structured interviews were conducted with 26 clinical pharmacists involved in antimicrobial reviews in intensive care and general medical units. Interviews incorporated the critical incident technique to elicit examples of accepted and rejected stewardship recommendations. Data were analyzed using a realist-informed approach to develop context–mechanism–outcome configurations explaining variations in pharmacists’ reported AMS contribution. Results: Pharmacotherapy expertise was necessary but not sufficient for pharmacists’ recommendations to shape antimicrobial prescribing. Leadership endorsement, structured multidisciplinary rounds, and formal documentation pathways activated mechanisms of legitimacy, credibility, and workflow visibility, supporting reported uptake of dose optimization, therapeutic drug monitoring, and selected de-escalation recommendations. In contrast, prescriber-led hierarchies, limited documentation pathways, workload pressures, and defensive prescribing cultures activated mechanisms of self-limitation, risk aversion, and limited recommendation uptake, particularly for discontinuation, duration control, and narrowing of broad-spectrum therapy. Conclusions: Strengthening AMS requires not only formal committees and guidelines but also team-based structures that integrate pharmacists’ pharmacotherapy expertise into antimicrobial review while preserving clinicians’ ultimate prescribing responsibility.

## 1. Introduction

Antimicrobial resistance (AMR) remains one of the most urgent global health challenges, with hospitals representing critical sites for antimicrobial exposure and resistance amplification [1]. Broad-spectrum initiation, combination therapy, and prolonged treatment durations are common in acutely ill patients, and inappropriate hospital prescribing contributes to adverse drug events, extended hospitalization, and escalating healthcare costs [2]. In response, antimicrobial stewardship (AMS) programs have become a central component of hospital quality improvement strategies worldwide [3].

Within hospital-based AMS programs, clinical pharmacists are increasingly recognized as key operational members of multidisciplinary stewardship teams [4,5,6]. Pharmacist-supported interventions, including prospective audit and feedback, de-escalation discussions, dose optimization, therapeutic drug monitoring, intravenous-to-oral conversion, and duration review, have been associated with improved prescribing quality and reduced unnecessary antimicrobial exposure [7,8]. In many settings, pharmacists are formally embedded within stewardship teams and contribute pharmacotherapy expertise to antimicrobial decision-making processes.

The decision to initiate, continue, escalate, narrow, or discontinue antimicrobial therapy remains the responsibility of the treating clinician, who integrates the patient’s history, clinical signs and symptoms, laboratory results, microbiological findings, and overall clinical risk. Clinical pharmacists do not replace this responsibility; rather, they support team-based AMS by contributing specialized pharmacotherapy input related to antimicrobial selection, dosing, monitoring, toxicity, de-escalation, and treatment duration.

However, stewardship effectiveness is not determined solely by individual clinical expertise or by the presence of formal AMS structures. Even where committees, policies, and guidelines are established, implementation outcomes vary considerably across institutions. Clinical pharmacists’ ability to translate pharmacotherapy expertise into practice is shaped by institutional culture, leadership signaling, documentation pathways, interprofessional dynamics, and tolerance for clinical uncertainty [9,10]. In hospital environments where prescribing decisions remain predominantly physician-led, pharmacists’ recommendations may be negotiated, accepted, modified, or not adopted depending on contextual conditions as well as clinical merit [11]. These dynamics are particularly relevant in low- and middle-income countries (LMICs), where AMS infrastructure is expanding but remains heterogeneous.

In Jordan, most Ministry of Health hospitals report the establishment of formal AMS committees, and clinical pharmacy practice has developed substantially over the past decade [12]. Nevertheless, stewardship implementation remains uneven across sectors and institutions, suggesting that formal governance structures alone do not guarantee consistent antimicrobial optimization. Existing research in the region has largely examined awareness, attitudes, or self-reported barriers to stewardship, particularly within community pharmacy settings [13,14]. Hospital-based studies have primarily been descriptive or cross-sectional, including evaluations of AMS core element compliance [12] and healthcare professionals’ perceptions of computerized decision-support systems [15].

Far less attention has been given to how antimicrobial prescribing decisions are negotiated in real time within multidisciplinary hospital teams and how contextual features enable or constrain the practical integration of pharmacists’ recommendations. Descriptive accounts of “barriers and facilitators” provide limited explanatory power regarding why stewardship contributions are incorporated into prescribing decisions in some contexts yet remain peripheral in others.

Implementation-oriented perspectives offer a way to better understand these dynamics. Realist approaches, for example, focus on explaining how contextual conditions activate mechanisms that produce particular outcomes within complex healthcare systems. Previous research has highlighted the importance of social and hierarchical influences on antimicrobial decision-making [16], yet few studies have applied such perspectives to examine pharmacist roles within physician-dominant hospital environments in LMICs.

Therefore, the present study aims to explain how contextual and interprofessional dynamics shape the integration of clinical pharmacists’ antimicrobial stewardship contributions within multidisciplinary prescribing decisions in Jordanian hospitals. By developing context–mechanism–outcome (CMO) configurations grounded in frontline clinical practice, the study seeks to generate an explanatory model of team-based stewardship contribution that may inform more effective implementation of hospital AMS programs in similar health system settings.

## 2. Results

### 2.1. Contextual Conditions Shaping Clinical Pharmacist Contributions to Team-Based AMS

Across participating hospitals, clinical pharmacists’ contribution to antimicrobial decision-making was strongly shaped by contextual conditions rather than by individual qualification alone. Pharmacotherapy expertise was consistently important, but its translation into prescribing decisions depended on how pharmacists were integrated into multidisciplinary workflows, documentation systems, and prescriber-led decision processes.

#### 2.1.1. Governance Structure and Leadership Signaling

In hospitals where medical directors or pharmacy leadership explicitly endorsed antimicrobial stewardship, pharmacists described greater legitimacy in raising recommendations. Leadership support functioned as a contextual enabler that signaled institutional expectation for collaborative, team-based antimicrobial review.

“*When the medical director clearly states that antimicrobial decisions should be reviewed with pharmacy, consultants take it seriously. It gives us confidence to speak*.” (CP15, ICU, Private hospital).

Conversely, in hospitals where AMS was present primarily to satisfy accreditation documentation, pharmacists reported symbolic recognition but limited integration into day-to-day prescribing workflows.

“*We have restriction policies on paper, but if the consultant insists, the antibiotic continues*.” (CP14, Clinical Pharmacist, MOH Hospital, Central Jordan).

In these contexts, stewardship policy existed formally but lacked behavioral reinforcement mechanisms.

#### 2.1.2. ICU Embedding as a Structural Context

Embedding within ICUs emerged as a critical contextual determinant. Pharmacists who attended daily ICU rounds reported higher visibility in antimicrobial discussions, while those limited to retrospective chart review described reactive rather than proactive stewardship roles.

“*In ICU, I am present during rounds every day. I present cultures and suggest narrowing therapy*.” (CP01, ICU, MOH).

However, ICU embedding alone did not guarantee decisional influence. In high-acuity settings, defensive prescribing practices frequently limited de-escalation, particularly when consultants prioritized perceived safety over microbiological guidance.

“*Even when cultures are negative, some consultants prefer to continue broad-spectrum antibiotics. The fear of deterioration is stronger than the guideline*.” (CP02, ICU, MOH).

Thus, ICU embedding created opportunity for influence but did not uniformly activate stewardship mechanisms.

#### 2.1.3. Documentation Pathways and Visibility in the Medical Record

The ability to formally document antimicrobial recommendations emerged as a structural differentiator. In hospitals where pharmacists could enter stewardship notes into electronic or paper charts, their recommendations were perceived as part of the clinical record and multidisciplinary care process rather than informal verbal suggestions.

“*When I document in the electronic system, it becomes part of the patient file. Doctors cannot ignore it easily*.” (CP16, Internal Medicine, Private).

In contrast, pharmacists restricted to verbal recommendations described limited follow-through, particularly during consultant handovers.

“*Sometimes I tell the resident, but if it’s not written officially, it disappears during shift change*.” (CP05, Internal Medicine, MOH).

Documentation pathways therefore functioned as contextual amplifiers of professional legitimacy and continuity of team-based review.

#### 2.1.4. Interprofessional Culture and Hierarchical Norms

Hospital culture influenced whether antimicrobial discussions were collaborative or strongly prescriber-led. In some institutions, residents were receptive to pharmacist input, whereas senior consultant decisions often shaped the boundaries within which recommendations could be discussed.

“*Residents are usually open to discussion. The challenge is when the consultant makes a decision before rounds and it’s difficult to reverse*.” (CP18, Internal Medicine, Private).

In consultant-dominant environments, pharmacists described moderating their approach to avoid professional tension.

“*You have to pick your battles. If you challenge everything, it creates tension*.” (CP12, ICU, MOH).

Hierarchical norms therefore limited how far pharmacist recommendations could be integrated into final prescribing decisions.

### 2.2. Mechanisms That Enable Uptake of Clinical Pharmacist Recommendations

While contextual factors created the structural environment for stewardship, uptake of pharmacist recommendations was ultimately shaped through identifiable mechanisms operating at the interpersonal and cognitive levels. These mechanisms explained why pharmacist input was sometimes incorporated into antimicrobial decisions and at other times remained advisory despite similar policies being in place.

#### 2.2.1. Credibility Through Repeated Clinical Accuracy

Trust did not emerge automatically from professional title; rather, it developed through repeated demonstration of accurate, patient-centered recommendations. Pharmacists described a gradual accumulation of “clinical credibility” that increased acceptance of antimicrobial suggestions over time.

“*In the beginning, they double-check everything you say. After some time, when they see that your dose adjustments and de-escalation suggestions are correct, they start asking you before prescribing*.” (CP03, ICU, MOH).

This mechanism functioned as relational capital. In hospitals where pharmacists had long-standing presence in specific units, stewardship interventions were more likely to be accepted without resistance.

Conversely, newly assigned pharmacists or rotating staff described reduced influence until credibility was re-established.

#### 2.2.2. Strategic Framing of Recommendations

Acceptance of stewardship advice was influenced by communication strategy. Pharmacists described framing recommendations around safety, evidence, or shared decision-making.

“*If I say ‘this antibiotic is unnecessary,’ it may cause tension. But if I say ‘creatinine is rising; we should consider narrowing therapy,’ they respond more positively*.” (CP20, Internal Medicine, Private).

“*When I show them our hospital antibiogram and explain resistance patterns, they usually reconsider*.” (CP07, ICU, MOH).

Strategic framing reduced perceived professional tension and enhanced uptake within multidisciplinary discussion.

#### 2.2.3. Timing Within Clinical Workflow

Timing emerged as a critical activation mechanism. Recommendations delivered during formal rounds were more likely to be accepted than those communicated retrospectively.

“*If I speak during rounds, the discussion happens in front of the team. But if I call later, it feels like questioning the decision*.” (CP04, ICU, MOH).

Pharmacists working on wards without structured round participation described limited opportunity to influence initial antibiotic choice, often intervening only after 48–72 h.

This suggests that temporal positioning within decision-making processes directly influenced stewardship effectiveness.

#### 2.2.4. Interdisciplinary Alignment as a Legitimizing Mechanism

In hospitals with infectious disease (ID) specialists, alignment with ID physicians enhanced pharmacist influence. When pharmacists and ID physicians presented unified recommendations, acceptance rates increased.

“*If ID supports the recommendation, it becomes easier. Alone, it may be ignored. Together, it carries more weight*.” (CP22, ICU, Private).

Thus, interdisciplinary alignment functioned as a legitimacy amplifier.

#### 2.2.5. Escalation Pathways and Institutional Backing

In some hospitals, pharmacists described having formal escalation mechanisms—such as reporting to pharmacy directors or AMS committees—when antimicrobial misuse persisted. This structural backup activated greater confidence in raising concerns.

“*Knowing that pharmacy leadership supports us makes it easier to insist when needed*.” (CP09, ICU, MOH).

Where such backing was absent, pharmacists often described self-limiting their interventions to avoid professional conflict.

### 2.3. Mechanisms That Limit or Neutralize Pharmacist Contributions

Despite supportive contexts in some institutions, participants consistently described mechanisms that limited or neutralized pharmacist contributions to team-based stewardship. These mechanisms operated at hierarchical, cultural, and psychological levels and could prevent appropriate pharmacotherapy expertise from being incorporated into final prescribing decisions.

#### 2.3.1. Prescriber-Led Decision Hierarchy and Limited Recommendation Uptake

A dominant limiting mechanism was prescriber-led decision hierarchy. In many hospitals, final antimicrobial decisions rested with senior consultants, and pharmacist recommendations could be accepted, modified, or not adopted depending on prescriber receptiveness and local documentation processes.

“*If the consultant decides to continue piperacillin-tazobactam, even without indication, nobody challenges it. We can suggest, but we cannot enforce*.” (CP06, Internal Medicine, MOH).

Prescriber-led hierarchy functioned as a structural boundary on the uptake of pharmacist recommendations.

#### 2.3.2. Defensive Prescribing Culture and Risk Aversion

In high-acuity settings, particularly ICUs, defensive prescribing emerged as a major suppressive mechanism. Physicians frequently prioritized perceived patient safety over guideline-directed de-escalation, particularly in unstable or elderly patients.

“*Even if cultures are negative, they say ‘what if something is missed?’ So antibiotics continue for safety*.” (CP10, ICU, MOH).

This culture of precaution was intensified by fear of clinical deterioration or medico-legal consequences. Pharmacists reported difficulty countering decisions driven by uncertainty.

“*Sometimes it is not about evidence. It is about fear*.” (CP24, ICU, Private).

In such contexts, stewardship mechanisms were weakened by risk-based reasoning.

#### 2.3.3. Dependence on Prescriber Approval for Discontinuation and Duration Control

Across several hospitals, pharmacists described being able to recommend dose adjustments but requiring prescriber approval for discontinuation or duration changes.

“*We can adjust dosing for renal function, but stopping an antibiotic requires physician approval*.” (CP08, Internal Medicine, MOH).

This asymmetry meant that stewardship often remained advisory rather than operational.

Even when de-escalation was clinically indicated, pharmacists described scenarios in which therapy continued due to inertia.

“*Once an antibiotic is started, it is rarely stopped early. It just continues until discharge*.” (CP25, Internal Medicine, Private).

Thus, the absence of structured stop or review mechanisms limited meaningful duration optimization.

#### 2.3.4. Workload and Role Dilution

Clinical pharmacists in MOH hospitals frequently reported balancing AMS with medication reconciliation, discharge counseling, drug information queries, and administrative tasks.

“*We are expected to do everything—clinical rounds, discharge summaries, medication errors. There is no protected time for stewardship*.” (CP11, ICU, MOH).

In these contexts, stewardship shifted from a proactive review during rounds to retrospective documentation, reducing opportunities for timely intervention. Role dilution therefore functioned as a systemic suppressor of influence.

### 2.4. Outcome Patterns of Stewardship Across Hospital Contexts

#### 2.4.1. Dose Optimization as a Low-Resistance Stewardship Domain

Across hospitals, dose adjustment—particularly in renal impairment—and therapeutic drug monitoring were described as the most consistently accepted stewardship interventions. These activities aligned closely with traditional pharmacy roles and were rarely perceived as encroaching on prescriber-led clinical decision-making.

As one pharmacist working in a public tertiary hospital explained:

“*When it comes to vancomycin levels or adjusting doses for creatinine clearance, doctors usually accept immediately. They see it as pharmacy expertise*.” (CP14, ICU, MOH).

Similarly, a participant from a private hospital noted:

“*Dose optimization is straightforward. It’s not challenging their clinical judgment; it’s improving safety*.” (CP17, Internal Medicine, Private).

In this domain, stewardship operated with minimal hierarchical resistance. However, several pharmacists emphasized that while dose optimization improved medication safety, it did not fundamentally alter antimicrobial spectrum or duration.

“*We can optimize the dose, but if the antibiotic itself is too broad, that’s a different discussion*.” (CP15, ICU, Private).

Thus, pharmacist contributions within dose optimization were technically significant but more limited for decisions regarding antimicrobial spectrum and duration, which remained primarily within prescriber-led clinical decision-making.

#### 2.4.2. De-Escalation Under Conditions of Evidentiary Legitimacy

De-escalation was described as achievable primarily when supported by clear microbiological evidence. Culture and sensitivity results functioned as legitimizing tools that reduced interpersonal tension and reframed narrowing therapy as data-driven rather than corrective.

An ICU pharmacist from a teaching hospital stated:

“*When cultures are positive and sensitivities are available, narrowing therapy becomes easier. You’re not arguing—you’re showing evidence*.” (CP02, ICU, MOH).

Another participant described presenting local antibiogram data during rounds:

“*If I show them resistance patterns from our hospital, they listen more. It becomes about the patient, not about who is right*.” (CP19, ICU, Private).

Evidence reduced interpersonal defensiveness and reframed narrowing as data-driven optimization.

#### 2.4.3. Discontinuation and Duration Control as Structurally Constrained Outcomes

Discontinuation and duration optimization were consistently described as the most difficult stewardship outcomes to achieve. Participants reported that once antimicrobials were initiated, particularly broad-spectrum agents, therapy frequently continued until discharge unless a formal review mechanism intervened. A clinical pharmacist reflected:

“*Stopping antibiotics is the hardest step. Even if the patient improves, nobody wants to take responsibility for stopping*.” (CP05, Internal Medicine, MOH).

Another participant described therapeutic inertia in the absence of systematic review:

“*If there is no system that forces review at 48 or 72 h, antibiotics just continue. It becomes routine*.” (CP16, Internal Medicine, Private).

Duration control failures reflected structural workflow gaps rather than lack of pharmacotherapy knowledge.

### 2.5. A Context–Mechanism–Outcome (CMO) Model of Clinical Pharmacist Contribution to Team-Based AMS

Synthesizing findings across hospitals, three recurrent Context–Mechanism–Outcome configurations were identified that explain variations in how clinical pharmacist contributions were integrated into team-based AMS. These configurations illustrate how institutional conditions activated or suppressed mechanisms of recommendation uptake, ultimately shaping reported antimicrobial decision-making patterns.

#### 2.5.1. Institutional Support and Structured Embedding Enable Routine Antimicrobial Optimization

In hospitals where antimicrobial stewardship was visibly endorsed by senior leadership and pharmacists were consistently embedded in ICU or multidisciplinary rounds, participants described a shift from optional advice to expected contribution within clinical reviews. Leadership signaling—whether through formal policy statements, AMS committee visibility, or direct support from medical directors—created a context in which pharmacist input was expected as part of team-based antimicrobial decision-making.

As one senior clinical pharmacist described:

“*When the medical director makes it clear that antimicrobial decisions should be discussed with pharmacy, consultants listen differently. It becomes part of the process*.” (CP09, ICU, MOH).

Within this context, pharmacists reported increased confidence in raising recommendations and were less hesitant to propose de-escalation or discontinuation.

“*If stewardship is supported by administration, you don’t feel like you’re interfering. You feel like you’re fulfilling your role*.” (CP22, ICU, Private).

Here, the contextual presence of leadership endorsement activated a mechanism of perceived legitimacy, reducing professional resistance and normalizing pharmacist participation in team-based reviews. The reported outcome was more routine antimicrobial review, perceived earlier narrowing of therapy, and greater scrutiny of broad-spectrum continuation.

#### 2.5.2. Consultant-Dominant Contexts Constrain Stewardship to Advisory Roles

In contrast, in hospitals characterized by strong consultant-centered decision-making and absence of formal documentation pathways, pharmacists described moderating their interventions. Even when clinically confident, they often framed recommendations cautiously or limited escalation to avoid professional tension.

One participant explained:

“*With some consultants, you have to be very careful. You can suggest, but you cannot insist*.” (CP12, ICU, MOH).

Another pharmacist reflected on selective intervention:

“*You learn which cases to push and which ones to leave. If you challenge everything, it creates tension*.” (CP26, Internal Medicine, Private).

In these contexts, lack of written documentation pathways further limited the continuity and uptake of pharmacist recommendations.

“*If I cannot document officially in the chart, the recommendation can disappear during shift change*.” (CP05, Internal Medicine, MOH).

The contextual dominance of prescriber-led decision-making activated a mechanism of self-limitation and strategic deference. Stewardship was reported to remain largely advisory, and de-escalation or discontinuation was described as occurring inconsistently, often dependent on physician receptiveness rather than systematic review.

#### 2.5.3. Clinical Uncertainty and Defensive Prescribing Sustain Broad-Spectrum Therapy

In high-acuity environments, particularly ICUs managing unstable patients, pharmacists described a culture of defensive prescribing. Even when microbiological data supported narrowing therapy, decisions were frequently influenced by perceived clinical risk.

As one ICU pharmacist stated:

“*In critical patients, even if cultures are negative, the consultant says, ‘Let’s continue for safety.’ It’s about avoiding risk*.” (CP02, ICU, MOH).

Another participant emphasized the emotional dimension of prescribing:

“*Sometimes it is not about guidelines; it is about fear of deterioration*.” (CP24, ICU, Private).

In these settings, clinical uncertainty activated risk aversion mechanisms that overrode evidence-based recommendations. The reported outcome was prolonged broad-spectrum coverage and delayed de-escalation, as perceived by participating pharmacists, despite pharmacist advocacy.

The three refined CMO configurations are summarized in Table 1 to illustrate how specific institutional contexts activated mechanisms that shaped pharmacists’ reported contributions to antimicrobial prescribing decisions within multidisciplinary teams.

## 3. Discussion

This realist-informed, multi-site qualitative study offers an explanatory account of how clinical pharmacists perceive and experience variations in the integration of their antimicrobial stewardship contributions within multidisciplinary prescribing decisions in Jordanian hospitals. The findings indicate that pharmacotherapy expertise is necessary but not sufficient for pharmacist recommendations to shape antimicrobial prescribing. Instead, pharmacists’ contributions are more likely to be incorporated into decision-making when institutional conditions activate mechanisms of legitimacy, credibility, and workflow integration, while prescriber-led hierarchy, clinical risk perception, and structural gaps may limit recommendation uptake. By identifying CMO configurations grounded in frontline clinical practice, the study provides an explanatory perspective on why AMS programs with similar formal structures may produce markedly different reported stewardship patterns across hospitals.

These findings should not be interpreted as suggesting that antimicrobial prescribing should shift from clinicians to pharmacists. Rather, they highlight that effective AMS depends on the integration of complementary professional expertise within multidisciplinary teams. Treating clinicians remain responsible for the overall decision to initiate, continue, escalate, narrow, or discontinue antimicrobial therapy, while clinical pharmacists contribute specialized input related to antimicrobial selection, dosing, monitoring, toxicity, de-escalation, and duration. This study therefore emphasizes team-based stewardship rather than professional competition for prescribing leadership.

A consistent pattern across sites was the differential uptake of pharmacist recommendations depending on how they aligned with established professional roles. Dose optimization and therapeutic drug monitoring were widely accepted and rarely contested, reflecting their close alignment with traditional pharmacy expertise. In contrast, recommendations involving antimicrobial spectrum narrowing, de-escalation, or discontinuation were more frequently negotiated or resisted. Similar observations have been reported internationally, where pharmacist-supported dosing interventions demonstrate higher acceptance rates than recommendations that alter initial prescribing decisions [6,17,18,19]. The present findings extend this understanding by suggesting that these differences reflect underlying professional boundary dynamics: stewardship activities perceived as improving treatment safety are more easily incorporated into clinical workflows than those perceived as modifying prescriber-led therapeutic strategies.

Leadership engagement emerged as a key contextual factor shaping the integration of pharmacist contributions. Pharmacists working in hospitals where stewardship activities were explicitly supported by medical directors or pharmacy leadership described greater confidence in raising recommendations and more consistent uptake of interventions. Organizational endorsement appeared to legitimize pharmacist participation in antimicrobial review, transforming stewardship from an optional consultative activity into an expected component of patient care. Previous implementation research has highlighted leadership engagement as a central determinant of AMS effectiveness [3,20,21]. The present findings suggest that leadership influence operates not only through governance structures but also through social signaling that reinforces pharmacists’ professional legitimacy within multidisciplinary teams.

The finding that “when the medical director makes it clear that antimicrobial decisions should be discussed with pharmacy, consultants listen differently” is particularly important. It suggests that sustainable AMS improvement requires explicit institutional endorsement and, where possible, national-level policy support. Clear hospital policies, leadership commitment, Ministry of Health recommendations, and standardized documentation pathways may help ensure that pharmacist participation in antimicrobial review is implemented consistently across hospitals rather than depending on individual prescriber receptiveness or local professional relationships.

Interprofessional hierarchy further shaped stewardship enactment. Although pharmacists reported constructive collaboration with resident physicians, senior consultant decisions frequently determined final antimicrobial treatment plans. In strongly prescriber-led environments, pharmacists described selectively moderating their recommendations to maintain professional relationships and avoid perceived confrontation. Such patterns are consistent with earlier qualitative studies demonstrating that antimicrobial prescribing decisions are embedded within professional hierarchies and social norms rather than guided exclusively by clinical guidelines [16,22]. Within this context, pharmacists often adopted strategic communication approaches, gradually building relational credibility through repeated demonstration of clinical accuracy. This mechanism of progressive trust-building highlights how pharmacist contributions can become more integrated over time but remain bounded by formal prescribing responsibility and local team culture.

Clinical uncertainty and defensive prescribing also influenced reported stewardship outcomes. In high-acuity environments such as intensive care units, physicians often preferred continued broad-spectrum therapy even when microbiological evidence supported de-escalation. Participants attributed this pattern to fear of clinical deterioration and perceived medico-legal risk associated with antimicrobial discontinuation. Previous studies have similarly highlighted the influence of uncertainty and risk perception on antimicrobial prescribing behavior [23,24]. The present findings suggest that defensive prescribing may function as a mechanism that overrides stewardship recommendations when clinicians perceive potential patient deterioration as a greater risk than antimicrobial overuse.

Documentation pathways emerged as another important determinant of sustained pharmacist contribution. Pharmacists who were able to document recommendations directly within electronic health records described greater continuity of stewardship interventions across shifts and consultant transitions. In contrast, verbal recommendations were more susceptible to omission or reinterpretation. Similar findings have been reported in studies examining the role of structured audit-and-feedback systems in antimicrobial stewardship [25,26,27]. These observations highlight how documentation functions not only as a technical process but also as an institutional mechanism that stabilizes stewardship recommendations within clinical workflows.

Together, these findings suggest that effective AMS depends on the alignment of several institutional conditions. Leadership endorsement legitimizes pharmacist participation, documentation systems preserve stewardship recommendations across clinical transitions, and collaborative professional cultures facilitate negotiation of antimicrobial decisions. When these enabling conditions are absent, pharmacist contributions remain advisory and vulnerable to non-uptake. This perspective helps explain why hospitals with similar AMS committees and policies may experience markedly different reported stewardship patterns. Our findings are also consistent with regional hospital data indicating that, even in institutions with antimicrobial stewardship structures in place, antibiotic utilization remains substantial and ongoing audit is needed; for example, a UAE point prevalence survey reported frequent use of broad-spectrum agents and 78.0% compliance with local/international guidelines, supporting the importance of continued stewardship monitoring beyond formal committee establishment [28].

Evidence from Jordan and neighboring Middle Eastern settings further supports the importance of context-sensitive AMS implementation. In Jordan, studies have reported formal progress in AMS core elements but ongoing barriers related to implementation consistency, healthcare provider awareness, and system-level support [12,29]. Qualitative work from Saudi hospitals similarly identified insufficient policy enforcement, fragmented teamwork, limited ASP staffing, communication gaps, and health-information technology constraints as barriers to AMS implementation [30], while regional survey evidence has emphasized the importance of leadership, training, and institutional support [31,32]. In Qatar, pharmacists described gaps between AMS education and real-world pharmacy practice, reinforcing the need for practice-based training and structured integration into clinical workflows [33]. These regional findings align with the present study by suggesting that pharmacist contribution to AMS depends on governance, workflow integration, and multidisciplinary collaboration rather than professional knowledge alone.

From an AMS implementation perspective, the findings suggest that hospitals should move beyond symbolic committee establishment and create operational conditions that integrate pharmacist contributions at the point of prescribing. These include formal documentation pathways for antimicrobial recommendations, pharmacist participation in 48–72 h antimicrobial review, pharmacist embedding in ICU and internal medicine rounds, and escalation pathways for unresolved inappropriate antimicrobial continuation. In addition, hospitals should address defensive prescribing through multidisciplinary case review, shared accountability for antimicrobial discontinuation, and local feedback using antibiograms, culture results, and antimicrobial-utilization data. Such strategies may help shift pharmacist involvement from optional advice to integrated team-based stewardship practice while maintaining clinicians’ ultimate prescribing responsibility.

## 4. Materials and Methods

### 4.1. Study Design and Philosophical Orientation

This study employed a multi-site, realist-informed qualitative design to examine how clinical pharmacists contribute to AMS within Jordanian hospital contexts. A realist-informed approach was selected to move beyond descriptive identification of barriers and facilitators and instead develop explanatory insights into how specific contextual conditions interact with underlying generative mechanisms to shape reported stewardship patterns. The analytic focus was on constructing and refining CMO configurations that explain variations in the integration and uptake of pharmacist recommendations across institutional settings. This study is reported in accordance with the Consolidated Criteria for Reporting Qualitative Research (COREQ) (Supplementary Material S1).

For the purpose of this study, “context” referred to the organizational, professional, and workflow conditions within which antimicrobial prescribing decisions occurred, including leadership support, documentation pathways, ward structure, multidisciplinary rounds, and interprofessional hierarchy. “Mechanisms” were conceptualized as the reasoning processes, relational responses, and professional dynamics activated by these contexts, such as perceived legitimacy, clinical credibility, risk aversion, self-limitation, and confidence to raise stewardship recommendations. “Outcomes” referred to reported patterns of pharmacist contribution to antimicrobial decision-making, including reported uptake of dose optimization, therapeutic drug monitoring, de-escalation, discontinuation, or continued broad-spectrum therapy.

The initial program theory guiding the analysis was that clinical pharmacists’ pharmacotherapy expertise is necessary but not sufficient to shape antimicrobial prescribing. Rather, pharmacists’ contributions are more likely to be integrated into team-based AMS when institutional and workflow conditions legitimize their input and embed their recommendations within clinical decision-making processes. Conversely, in contexts characterized by strong prescriber-led hierarchy, limited documentation pathways, or high clinical uncertainty, pharmacists’ contributions may remain advisory despite appropriate clinical knowledge. This initial program theory was iteratively refined through critical incident narratives, retroductive reasoning, and cross-case comparison across hospital settings.

### 4.2. Study Setting and Site Selection

This study was conducted across multiple Jordanian hospitals purposively selected to capture variations in antimicrobial stewardship infrastructure and clinical pharmacy practice models. Participating sites included Ministry of Health hospitals and private or teaching hospitals distributed across different regions of Jordan. Hospitals differed in AMS maturity, including the presence or absence of formal stewardship committees, infectious diseases specialist support, antimicrobial restriction policies, documentation pathways, and structured audit-and-feedback processes. This variation allowed systematic cross-context comparison of pharmacists’ team-based stewardship contributions under differing organizational conditions.

### 4.3. Participants and Eligibility Criteria

Participants were licensed pharmacists working in hospital-based clinical roles with direct involvement in antimicrobial decision-making. Eligible participants included clinical pharmacists and PharmD practitioners (pharmacists holding a Doctor of Pharmacy degree and practicing in hospital-based clinical roles) who routinely participated in ICU or ward rounds, antimicrobial review, dose optimization, therapeutic drug monitoring, de-escalation discussions, intravenous-to-oral conversion, or documentation of antimicrobial recommendations where applicable. Pharmacists whose responsibilities were limited exclusively to dispensing or administrative tasks without routine engagement in antimicrobial clinical decisions were excluded.

### 4.4. Sampling Strategy and Sample Adequacy

A purposive maximum-variation sampling strategy was employed to capture diversity across hospital sectors, geographic regions, clinical settings, and years of professional experience. Sampling also incorporated deviant case selection to include pharmacists reporting consistently high influence and those describing limited influence in antimicrobial decision-making.

Sample adequacy was guided by the principle of information power and by realist sufficiency criteria. Recruitment continued until the dataset enabled stable and coherent development of CMO configurations capable of explaining variations across institutional contexts. Decisions to cease recruitment were based on explanatory completeness rather than frequency-based thematic saturation alone.

### 4.5. Recruitment and Consent Procedures

Potential participants were identified through clinical pharmacy departments and professional networks. Invitations were distributed via social media platforms or through departmental contact points and accompanied by an information sheet detailing the study purpose, voluntary nature of participation, confidentiality safeguards, and right to withdraw. Verbal informed consent was obtained prior to interviews. Verbal consent was used because the study involved minimal-risk qualitative interviews, and avoiding signed consent forms reduced the collection of directly identifiable documentation. The verbal consent procedure was approved by the Institutional Review Board. Participation was independent of employment evaluation to minimize perceived coercion. A total of 32 eligible pharmacists were approached; 26 agreed to participate, while 6 declined or did not respond. The main reasons for non-participation were limited time, scheduling constraints, or lack of availability.

Participating hospitals were approached through clinical pharmacy departments or departmental gatekeepers. Interviews were conducted only after institutional permission had been obtained in accordance with the administrative requirements of each participating site. No patient data were collected, and the study did not involve any intervention in clinical care.

### 4.6. Data Collection

#### 4.6.1. Interview Guide Development

The semi-structured interview guide was developed to explore clinical pharmacists’ experiences with antimicrobial stewardship decision-making in hospital settings. The guide focused on pharmacists’ participation in antimicrobial review, interactions with prescribers during clinical rounds, and contextual factors influencing the uptake of stewardship recommendations. Development of the interview questions was informed by previous qualitative and implementation research examining antimicrobial stewardship practices and interprofessional dynamics in hospital care [10,34,35]. Although these studies were conducted in different healthcare contexts, they were used as sensitizing references for general AMS and interprofessional concepts rather than as direct templates. The draft interview guide was reviewed by two Jordanian clinical pharmacy academics with AMS expertise to ensure clarity, cultural appropriateness, and relevance to local hospital workflows, prescriber-led decision structures, documentation practices, and availability of infectious diseases support. Minor revisions were made to improve question flow and ensure that the prompts elicited detailed experiential narratives relevant to Jordanian AMS practice. The final English-language version of the interview guide is provided in Supplementary Material S2.

#### 4.6.2. Interview Approach

Data were collected between August and November 2025 through semi-structured, in-depth interviews lasting approximately 45 to 60 min. Interviews were conducted in Arabic and were held face-to-face in a private room within or near the participants’ workplace, according to participant preference and scheduling feasibility. No non-participants were present during the interviews. The interview guide explored antimicrobial decision-making processes, interprofessional negotiation dynamics, leadership and governance influences, documentation practices, and experiences of recommendation uptake or non-uptake. No repeat interviews were conducted.

#### 4.6.3. Realist Elicitation and Critical Incident Technique

To support realist explanation-building, interviews incorporated a critical incident technique. Participants were asked to describe specific recent cases in which their recommendations contributed to antimicrobial therapy decisions and cases in which their recommendations were not adopted. These case-based narratives enabled identification of contextual triggers, underlying reasoning processes, and reported decision-making outcomes. Interview prompts were designed to elicit both descriptive and explanatory accounts, allowing inference of candidate mechanisms operating within specific organizational contexts.

#### 4.6.4. Recording, Transcription, and Translation

With participant consent, interviews were audio-recorded and transcribed verbatim. Field notes were recorded immediately after interviews to document contextual observations, initial analytic impressions, and reflexive considerations. Interviews were translated into English for analysis and reporting. A bilingual investigator reviewed a subset of transcripts and translations to ensure conceptual accuracy, particularly regarding terminology related to hierarchy, role integration, and clinical negotiation. Translation decisions were documented within the audit trail.

### 4.7. Data Analysis

Data analysis followed an iterative realist-informed process. Initial open coding was conducted to identify recurring patterns within participant narratives. These codes were then examined through retroductive reasoning to infer underlying mechanisms operating within specific institutional contexts. Through cross-case comparison, codes and mechanisms were integrated into CMO configurations explaining variations in pharmacists’ reported contribution to antimicrobial stewardship. A coding tree illustrating the relationship between initial open codes, interpretive categories, inferred mechanisms, and final analytical domains used to construct the CMO configurations is provided in Supplementary Material S3.

To distinguish CMO configurations from descriptive themes, the analytic team explicitly separated contextual conditions, inferred mechanisms, and reported stewardship patterns during coding and memo-writing. For example, the presence of structured multidisciplinary rounds was coded as a contextual condition, increased confidence to raise antimicrobial recommendations was interpreted as a mechanism, and reported uptake of dose optimization or de-escalation was coded as an outcome pattern. Candidate CMO configurations were first developed by the primary analyst and then discussed with the wider research team to assess coherence, plausibility, and fit with deviant cases. Disagreements were resolved through discussion and re-examination of relevant transcript excerpts until consensus was reached.

#### 4.7.1. Initial Coding and Familiarization

Transcripts were read repeatedly to achieve immersion in the data. A hybrid coding framework was developed, combining inductive codes grounded in participant narratives with sensitizing concepts drawn from implementation science to systematically attend to organizational and workflow determinants. Coding at this stage remained closely tied to participant language to avoid premature theoretical abstraction.

#### 4.7.2. Development of Candidate Program Theories

Analytic memos were used to articulate provisional explanatory propositions regarding how pharmacist contributions to stewardship operate in practice. These candidate program theories were framed as tentative CMO propositions linking institutional conditions with inferred generative mechanisms and reported antimicrobial decision-making patterns.

#### 4.7.3. Retroductive Mechanism Identification

Retroductive reasoning was applied to infer the most plausible underlying mechanisms generating observed patterns. Mechanisms were conceptualized as reasoning processes, relational dynamics, and cognitive responses triggered under specific contextual conditions. Competing interpretations were examined to ensure conceptual coherence and analytic rigor.

#### 4.7.4. Cross-Case Comparison and CMO Refinement

Cross-case matrices were constructed to compare patterns across hospital types and clinical settings. Contextual configurations were mapped alongside reported stewardship patterns to identify recurring explanatory relationships. CMO configurations were iteratively refined through constant comparison and examination of deviant cases until explanatory stability and coherence were achieved. Qualitative data management was supported using NVivo software (Version 12). An audit trail documented analytic decisions, coding iterations, and theory refinement.

### 4.8. Research Team, Reflexivity, and Positionality

Interviews were conducted by a trained male, PhD-level clinical pharmacist and academic researcher with clinical training and prior experience in qualitative interviewing. At the time of the study, the interviewer was working as a faculty member and clinical pharmacy academic. The interviewer was not involved in participants’ supervision, employment, or professional evaluation. Although some participants were known to the interviewer through professional healthcare networks, no formal researcher–participant relationship was established specifically for the study prior to recruitment, beyond existing professional familiarity in some cases. At the beginning of each interview, the researcher introduced himself in his role as a researcher and clarified the purpose of the study to minimize potential power dynamics and encourage open and candid discussion. Reflexive memos documented assumptions, positionality considerations, and interpretive decisions throughout data collection and analysis. Team discussions were used to examine alternative explanations and mitigate interpretive bias.

### 4.9. Trustworthiness and Rigor

Credibility, dependability, confirmability, and transferability were enhanced through several complementary strategies. Two researchers independently coded an initial subset of transcripts to calibrate the coding framework and resolve interpretive discrepancies before proceeding with full analysis. Deviant case analysis was applied throughout the analytic process to refine emerging explanations and ensure that alternative interpretations were considered. To enhance interpretive validity, a brief summary of the preliminary explanatory propositions was shared with a subset of participants to assess resonance with their experiences. Full interview transcripts were not returned for correction in order to minimize respondent burden. Detailed contextual descriptions were maintained to support transferability while preserving participant confidentiality. Analytic transparency was strengthened through maintenance of an audit trail and reflexive documentation throughout data collection and analysis.

### 4.10. Ethics Approval and Consent to Participate

Ethical approval was obtained from the Institutional Review Board at Al-Ahliyya Amman University (IRB No. AAU/4/6/2024–2025; approved on 3 July 2025) prior to study commencement. This study was conducted in accordance with the principles of the Declaration of Helsinki. All participants provided verbal informed consent before interviews. Identifying details were removed during transcription to protect confidentiality. Data were stored securely and accessed only by the research team.

### 4.11. Participant and Institutional Characteristics

A total of 26 clinical pharmacists were recruited from 9 hospitals across Jordan, including Ministry of Health hospitals and private or teaching hospitals located in Amman, Irbid, Zarqa, and southern Jordan. Within each hospital, purposive sampling was used to recruit pharmacists directly involved in antimicrobial decision-making and stewardship activities.

Participants had between 2 and 17 years of clinical experience (median 8 years). All held a PharmD degree or postgraduate clinical pharmacy qualification and were actively engaged in antimicrobial optimization within ICU and general medical settings.

Clinical embedding varied by ward type. Thirteen participants were primarily assigned to ICU services and reported routine daily participation in multidisciplinary rounds. Eight were assigned to internal medicine wards, where structured rounds occurred regularly but coverage intensity varied depending on staffing. Five described cross-coverage models across multiple wards.

Institutional antimicrobial stewardship governance also varied across sites. Seven of the nine hospitals reported having formally established antimicrobial stewardship (AMS) committees, consistent with national Ministry of Health data indicating widespread committee establishment in public hospitals. Two hospitals described stewardship activities occurring within routine clinical workflows without a formally constituted multidisciplinary AMS committee structure.

Across sites with formal committees, variability was observed in committee functionality, infectious diseases specialist involvement, restriction policies, and frequency of audit-and-feedback processes. These contextual differences were deliberately sampled to enable realist-informed comparative analysis of how governance structures influenced stewardship mechanisms and outcomes.

Documentation pathways also differed across institutions, with pharmacists in some hospitals able to enter recommendations directly into the electronic medical record, while others relied on pharmacy notes and verbal communication during rounds.

Participant and institutional characteristics are summarized in Table 2.

## 5. Limitations

Several limitations should be considered when interpreting the findings of this study. First, this study was based on the perspectives of clinical pharmacists only. Although pharmacists are central actors in antimicrobial stewardship and were able to provide detailed accounts of stewardship negotiation in daily practice, this study did not include physicians, residents, infectious disease specialists, nurses, hospital administrators, or members of antimicrobial stewardship committees. Therefore, the findings represent pharmacists’ perceived and experienced influence on antimicrobial decision-making rather than a complete interprofessional account of antimicrobial prescribing processes. Future studies incorporating prescribers and other stakeholders would provide a more comprehensive understanding of how pharmacist recommendations are interpreted, accepted, modified, or rejected within multidisciplinary teams.

Second, this study relied on self-reported interview narratives rather than direct observation of clinical rounds or objective antimicrobial prescribing data. Participants described examples of accepted and rejected recommendations, de-escalation, discontinuation, broad-spectrum continuation, and therapeutic drug monitoring; however, these reported stewardship patterns were not independently verified through chart review, prescribing audits, antimicrobial consumption indicators, or clinical outcome measures. As a result, the findings should be interpreted as explanatory accounts of perceived pharmacist contribution and decision-making patterns rather than measured effects on antibiotic use, resistance rates, length of stay, or patient outcomes. This study was not designed to estimate the objective impact of pharmacist involvement on antimicrobial utilization or clinical outcomes; future mixed-methods studies should combine qualitative analysis with prescribing audits, intervention acceptance rates, antimicrobial consumption metrics, and patient-level outcomes.

Third, although the use of critical incident technique encouraged participants to provide concrete examples from practice, recall bias may have influenced the selection and detail of the incidents described. Participants may have been more likely to remember unusual, difficult, or professionally meaningful cases, while routine interactions may have been underreported. In addition, social desirability bias cannot be excluded, as participants may have emphasized successful interventions, professional competence, or institutional challenges in ways that reflected their own interpretation of stewardship practice.

Fourth, the interviewer was a clinical pharmacy academic with professional familiarity with some participants through healthcare networks. Although there was no direct supervisory or employment relationship, this professional proximity may have influenced how openly participants discussed interprofessional tensions, institutional weaknesses, or limitations in their own practice. To reduce this risk, the researcher clarified the voluntary nature of participation, emphasized confidentiality, and maintained reflexive memos throughout data collection and analysis. Nevertheless, the possibility of interviewer–participant influence should be acknowledged.

Fifth, interviews were conducted in Arabic and translated into English for analysis and reporting. Although bilingual review was used to preserve conceptual accuracy, especially for terms related to hierarchy, role integration, professional negotiation, and clinical uncertainty, some nuances may have been altered during translation. This is particularly relevant in qualitative research where participants’ wording, tone, and culturally embedded expressions may carry interpretive meaning. Translation decisions were documented to strengthen transparency, but complete equivalence between Arabic narratives and English presentation cannot be guaranteed.

Sixth, this study included 26 clinical pharmacists from nine hospitals across different sectors and regions of Jordan, which strengthened the contextual variation; however, the findings may not be transferable to all Jordanian hospitals or to other healthcare systems. Hospitals with different staffing models, prescribing cultures, infectious disease support, electronic medical record systems, antimicrobial restriction policies, or levels of clinical pharmacy integration may activate different mechanisms of pharmacist contribution. Therefore, transferability should be judged according to similarity in antimicrobial stewardship maturity, professional hierarchy, documentation pathways, and clinical pharmacy workforce capacity.

Finally, because this was a realist-informed qualitative study rather than a full realist evaluation, the context–mechanism–outcome configurations developed here should be considered explanatory propositions rather than definitive causal claims. The configurations provide a theoretically informed model of how pharmacist contributions to team-based AMS may be activated or suppressed in physician-centered hospital settings, but they require further testing and refinement in future studies. Mixed-methods research combining qualitative observation, physician interviews, prescribing audits, and antimicrobial-use indicators could strengthen the empirical assessment of these mechanisms and determine whether the identified institutional conditions are associated with measurable improvements in antimicrobial prescribing.

## 6. Conclusions

Overall, this study contributes a realist-informed explanatory model of how clinical pharmacists’ contributions to team-based AMS are perceived to emerge within hospital practice. The findings demonstrate that strengthening antimicrobial stewardship requires more than establishing formal committees or guidelines; it also requires institutional conditions that legitimize pharmacists’ participation, embed their recommendations within clinical workflows, preserve clinicians’ prescribing responsibility, and address defensive prescribing cultures. By clarifying the contextual triggers that enable or constrain the uptake of pharmacist recommendations, this work provides practical insight for optimizing multidisciplinary antimicrobial stewardship in Jordan and comparable health system environments. In addition, consistent institutional leadership and national-level stewardship guidance may help standardize the integration of pharmacists’ contributions across hospitals.

## Figures and Tables

**Table 1:** Refined Context–Mechanism–Outcome configurations of clinical pharmacist contribution to team-based AMS.

Context	Mechanism Activated	Reported Stewardship Pattern
Leadership endorsement, structured multidisciplinary rounds, formal documentation pathways, and pharmacist embedding in ICU/ward workflows	Perceived legitimacy, confidence to raise recommendations, visibility in decision-making, and accumulated clinical credibility	More routine antimicrobial review, greater uptake of dose optimization and therapeutic drug monitoring, and more feasible de-escalation when supported by microbiological evidence
Prescriber-led decision hierarchy, limited documentation pathways, and weak escalation processes	Self-limitation, strategic deference, reduced perceived role integration, and concern about professional tension	Pharmacist role remains largely advisory; recommendations depend on individual physician receptiveness; de-escalation and discontinuation occur inconsistently
High-acuity clinical uncertainty, fear of deterioration, and defensive prescribing norms	Risk aversion, precautionary reasoning, reluctance to stop therapy, and preference for maintaining broad-spectrum coverage	Prolonged broad-spectrum antimicrobial therapy and delayed narrowing or discontinuation, despite pharmacist recommendations

**Table 2:** Participant and institutional characteristics (*n* = 26).

Characteristic	*n* (%)
Hospital Sector	
Ministry of Health	14 (53.8)
Private/Teaching	12 (46.2)
Hospitals with Formal AMS Committee	
Yes	7 of 9
No	2 of 9
Primary Clinical Assignment	
Intensive Care Unit (ICU)	13 (50.0)
Internal Medicine Ward	8 (30.8)
Rotational/Cross-Ward Coverage	5 (19.2)
Years of Clinical Experience	
<5 years	9 (34.6)
5–10 years	10 (38.5)
>10 years	7 (26.9)
Routine Participation in Multidisciplinary Rounds	
Daily (ICU or Ward-Based)	18 (69.2)
Regular but Not Daily	8 (30.8)
Documentation Modality	
Direct EMR documentation	13 (50.0)
Pharmacy note and/or verbal during rounds	13 (50.0)

Note. AMS = Antimicrobial Stewardship; ICU = Intensive Care Unit; EMR = Electronic Medical Records. Percentages were calculated using the total sample size (*n* = 26).

## Data Availability

Due to the qualitative nature of the study and the need to protect participant confidentiality, full interview transcripts and potentially identifiable data are not publicly available. Relevant supporting materials are provided in the article and Supplementary Materials. Further inquiries may be directed to the corresponding author upon reasonable request.

## Supplementary Materials

The following supporting information can be downloaded at: https://www.mdpi.com/article/10.3390/antibiotics15070670/s1, Supplementary Material S1: Completed COREQ checklist; Supplementary Material S2: Semi-structured interview guide; Supplementary Material S3: Coding Tree for the Realist-Informed Analysis.

## Abbreviations

The following abbreviations are used in this manuscript:

AMR	Antimicrobial Resistance
AMS	Antimicrobial Stewardship
CMO	Context–Mechanism–Outcome
COREQ	Consolidated Criteria for Reporting Qualitative Research
EMR	Electronic Medical Record
ICU	Intensive Care Unit
ID	Infectious Diseases
LMICs	Low- and Middle-Income Countries
MOH	Ministry of Health
PharmD	Doctor of Pharmacy
TDM	Therapeutic Drug Monitoring
