# Supplementary material for: Contextual Determinants of Clinical Pharmacists’ Contributions to Team-Based Antimicrobial Stewardship in Jordanian Hospitals: A Realist-Informed Qualitative Study
Source: Antibiotics (Basel). 2026 Jul 8;15(7):670. doi: 10.3390/antibiotics15070670 (PMC13406008; doi:10.3390/antibiotics15070670)
Supplement: Supplementary file 1 [file antibiotics-15-00670-s001.zip › COREQ_Checklist_Supplementary S1.pdf]

Supplementary Table S1. COREQ (32-item) Checklist

Determinants of clinical pharmacists' influence on antimicrobial prescribing in Jordanian hospitals: A Realist-Informed qualitative study

Domain	Item	How addressed in the manuscript
Domain 1: Research team and reflexivity	1. Interviewer/facilitator	Interviews were conducted by a trained male, PhD-level clinical pharmacist and academic researcher.
Domain 1: Research team and reflexivity	2. Credentials	Interviewer described as a PhD-level clinical pharmacist and academic researcher.
Domain 1: Research team and reflexivity	3. Occupation	Academic researcher and clinical pharmacist.
Domain 1: Research team and reflexivity	4. Gender	Interviewer identified as male.
Domain 1: Research team and reflexivity	5. Experience and training	Prior experience in qualitative interviewing and clinical training reported.
Domain 1: Research team and reflexivity	6. Relationship established	Some participants were known through professional healthcare networks; no direct hierarchical or dependent relationships existed.
Domain 1: Research team and reflexivity	7. Participant knowledge of the interviewer	At interview start, the researcher introduced himself in his role as a researcher and clarified the purpose of the study.
Domain 1: Research team and reflexivity	8. Interviewer characteristics	Reflexive memos documented assumptions, positionality considerations, and interpretive decisions; team discussions examined alternative explanations.
Domain 2: Study design	9. Methodological orientation and theory	Multi-site, realist-informed qualitative design focused on context-mechanism-outcome configurations.
Domain 2: Study design	10. Sampling	Purposive maximum-variation sampling with deviant case selection; purposive recruitment also described in participant characteristics section.
Domain 2: Study design	11. Method of approach	Potential participants were identified through clinical pharmacy departments and professional networks; invitations were distributed via social media platforms or departmental contact points.
Domain 2: Study design	12. Sample size	Abstract reports 26 clinical pharmacists; participant characteristics section reports 26 pharmacists from 9 hospitals.
Domain 2: Study design	13. Non-participation	A total of 32 eligible pharmacists were approached; 26 agreed to participate, while 6 declined or did not respond. The main reasons for non-participation were limited time, scheduling constraints, or lack of availability.
Domain 2: Study design	14. Setting of data collection	Study conducted across nine Jordanian hospitals; interviews explored hospital antimicrobial decision-making. Exact physical interview location was not specified.
Domain 2: Study design	15. Presence of non-participants	No non-participants were present during the interviews.

Domain 2: Study design	16. Description of sample	Hospital sector, ward assignment, years of experience, routine rounds participation, and documentation modality are summarized in text and Table 1.
Domain 2: Study design	17. Interview guide	Semi-structured interview guide described; guide development and expert review reported. Pilot testing not specifically reported.
Domain 2: Study design	18. Repeat interviews	No repeat interviews were conducted
Domain 2: Study design	19. Audio/visual recording	Interviews were audio-recorded with participant consent.
Domain 2: Study design	20. Field notes	Field notes were recorded immediately after interviews
Domain 2: Study design	21. Duration	Interviews lasted approximately 45 to 60 minutes.
Domain 2: Study design	22. Data saturation	Sample adequacy was guided by information power and realist sufficiency rather than frequency-based thematic saturation alone.
Domain 2: Study design	23. Transcripts returned	Full interview transcripts were not returned for correction to minimize respondent burden.
Domain 3: Analysis and findings	24. Number of data coders	Two researchers independently coded an initial subset of transcripts to calibrate the coding framework.
Domain 3: Analysis and findings	25. Description of the coding tree	Coding tree provided as supplementary material describing the hierarchical coding framework used in the realist-informed analysis.
Domain 3: Analysis and findings	26. Derivation of themes	Hybrid approach: initial open coding and inductive codes grounded in narratives, alongside sensitizing concepts from implementation science.
Domain 3: Analysis and findings	27. Software	NVivo software (Version 12) was used.
Domain 3: Analysis and findings	28. Participant checking	A brief summary of preliminary explanatory propositions was shared with a subset of participants to assess resonance.
Domain 3: Analysis and findings	29. Quotations presented	Extensive quotations are presented throughout Results and are identified using participant IDs and practice settings.
Domain 3: Analysis and findings	30. Data and findings consistent	Results present quotations aligned with each explanatory claim, and Discussion/Conclusion remain consistent with reported findings.
Domain 3: Analysis and findings	31. Clarity of major themes	Major themes are clearly structured as contextual conditions, activating/suppressing mechanisms, outcome patterns, and CMO configurations.
Domain 3: Analysis and findings	32. Clarity of minor themes	Deviant case sampling and analysis are reported, and the Results discuss variation across hospital types, units, and governance contexts.

Abbreviation: COREQ = Consolidated Criteria for Reporting Qualitative Research.
