# Supplementary material for: Contextual Determinants of Clinical Pharmacists’ Contributions to Team-Based Antimicrobial Stewardship in Jordanian Hospitals: A Realist-Informed Qualitative Study
Source: Antibiotics (Basel). 2026 Jul 8;15(7):670. doi: 10.3390/antibiotics15070670 (PMC13406008; doi:10.3390/antibiotics15070670)
Supplement: Supplementary file 1 [file antibiotics-15-00670-s001.zip › Interview guide Supplementary Material S2.pdf]

Clinical Pharmacists' Influence on Antimicrobial Stewardship in Jordanian Hospitals: A Realist-Informed Qualitative Study

Supplementary Material S2 – Semi-Structured Interview Guide

Introduction to Participants

Participants were informed that the purpose of the interview was to explore their experiences with antimicrobial stewardship (AMS) in hospital practice. They were assured that participation was voluntary, responses would remain confidential, and no identifying information would be reported.

Interview Questions

1. Professional Background

1. Can you describe your current role as a clinical pharmacist in your hospital?
2. How long have you been practicing in clinical pharmacy?
3. In which clinical units do you usually participate in patient care (e.g., ICU, internal medicine)?

---

2. Antimicrobial Stewardship Practice

4. Can you describe how antimicrobial prescribing decisions are typically made in your hospital?
5. What role do you play in reviewing antimicrobial therapy?
6. During clinical rounds, how are antimicrobial treatments discussed?

---

3. Influence on Antimicrobial Decisions

7. Can you describe a situation where you recommended a change to an antimicrobial therapy?
8. What happened after your recommendation?
9. What factors influenced whether your recommendation was accepted or not?

---

4. Interprofessional Dynamics

10. How do you usually communicate antimicrobial-related recommendations to physicians?
11. Do you notice differences in how residents and consultants respond to your recommendations?
12. How do hierarchical relationships influence antimicrobial decision-making?

---

5. Documentation and Workflow

13. Do you have the ability to document your recommendations in the patient's medical record?
14. How does documentation affect whether your recommendations are followed?
15. Are there systems or policies that support antimicrobial stewardship in your hospital?

---

6. Clinical Uncertainty and Prescribing

16. How are antimicrobial decisions handled when microbiological results are unavailable or unclear?
17. In your experience, how does clinical uncertainty influence prescribing decisions?

---

7. Institutional Context

18. How does hospital leadership influence antimicrobial stewardship practices?
19. What factors make it easier or more difficult for you to influence antimicrobial prescribing?

---

8. Reflections

20. What changes would improve antimicrobial stewardship in your hospital?
21. What would strengthen the role of clinical pharmacists in antimicrobial decision-making?

Closing Question

22. Is there anything else about antimicrobial stewardship in your hospital that you think is important?
