# Supplementary material for: Contextual Determinants of Clinical Pharmacists’ Contributions to Team-Based Antimicrobial Stewardship in Jordanian Hospitals: A Realist-Informed Qualitative Study
Source: Antibiotics (Basel). 2026 Jul 8;15(7):670. doi: 10.3390/antibiotics15070670 (PMC13406008; doi:10.3390/antibiotics15070670)
Supplement: Supplementary file 1 [file antibiotics-15-00670-s001.zip › Supplementary Material S3.pdf]

# Supplementary Material S3

## Coding Tree for the Realist-Informed Analysis

This supplementary material provides the coding tree used to support the realist-informed analysis. The table illustrates how initial open codes derived from participants' narratives were grouped into interpretive categories, how these categories informed the identification of mechanisms, and how the final CMO configurations were developed. The coding tree is intended to enhance analytic transparency rather than present a quantitative frequency count of codes.

**Abbreviations:** AMS, antimicrobial stewardship; CMO, context–mechanism–outcome; EMR, electronic medical record; ICU, intensive care unit; ID, infectious diseases; TDM, therapeutic drug monitoring.

### Table S3.1. Summary coding tree linking open codes to final CMO configurations

| Analytical domain | Examples of initial open codes | Interpretive categories | Inferred mechanisms | Final CMO configuration / reported stewardship outcome pattern |
|---|---|---|---|---|
| Institutional governance and workflow embedding | Leadership support; medical director endorsement; visible pharmacy leadership; formal AMS committee activity; structured multidisciplinary rounds; daily ICU participation; pharmacist present before decisions are finalized; direct EMR documentation; official chart note; escalation to pharmacy director or AMS committee. | Visible institutional mandate; pharmacist role formally embedded in workflow; recommendation visibility and continuity; organizational backing for stewardship; clinical pharmacy presence normalized within antimicrobial review. | Perceived legitimacy; confidence to raise recommendations; visibility in decision-making; accumulated clinical credibility; reduced hesitation to challenge inappropriate antimicrobial continuation. | CMO configuration 1: Institutional support and structured embedding enable routine antimicrobial optimization. Reported outcome pattern: more routine antimicrobial review, greater acceptance of dose optimization and TDM, and more feasible de-escalation when supported by microbiological evidence. |
| Interprofessional hierarchy and authority boundaries | Consultant insists on continuing therapy; resident receptive but cannot overrule consultant; pharmacist can suggest but not enforce; "pick your battles"; recommendation disappears during handover; lack of official documentation authority; weak escalation pathway; reluctance to create tension. | Consultant-dominant prescribing culture; pharmacist influence dependent on individual physician receptiveness; advisory rather than operational authority; fragile interprofessional negotiation; professional boundary management. | Self-limitation; strategic deference; reduced perceived authority; concern about professional tension; selective intervention; avoidance of repeated confrontation. | CMO configuration 2: Consultant-dominant contexts constrain stewardship to advisory roles. Reported outcome pattern: recommendations are inconsistently adopted, and de-escalation or discontinuation depends heavily on prescriber receptiveness rather than systematic review. |
| Clinical uncertainty and defensive prescribing culture | Cultures negative but antibiotic continued; "continue for safety"; fear of deterioration; unstable ICU patient; elderly or high-risk patient; broad-spectrum therapy maintained despite evidence; reluctance to stop antimicrobial therapy; concern about medico-legal or clinical consequences. | Low tolerance for uncertainty; precautionary prescribing norms; perceived risk of discontinuation exceeds perceived risk of antimicrobial overuse; defensive continuation of broad-spectrum therapy. | Risk aversion; precautionary reasoning; reluctance to stop therapy; preference for maintaining broad-spectrum coverage; fear-driven decision-making overriding evidence-based recommendations. | CMO configuration 3: Clinical uncertainty and defensive prescribing sustain broad-spectrum antimicrobial use. Reported outcome pattern: prolonged broad-spectrum therapy and delayed narrowing or discontinuation despite pharmacist recommendations. |
| Role-congruent stewardship activities versus contested prescribing decisions | Renal dose adjustment accepted; vancomycin levels reviewed; TDM recommendations accepted quickly; dose optimization seen as pharmacy expertise; narrowing therapy or stopping antibiotics described as "a different discussion"; antimicrobial spectrum and duration remain physician-controlled. | Distinction between low-resistance and high-resistance stewardship domains; safety-oriented interventions viewed as pharmacist-aligned; spectrum and duration decisions viewed as physician-led therapeutic authority. | Professional role alignment; reduced threat to prescriber authority; credibility through accurate technical recommendations; boundary protection around core prescribing decisions. | Cross-cutting explanatory pattern: dose optimization and TDM are accepted more readily than de-escalation or discontinuation. Reported outcome pattern: pharmacists achieve consistent influence in technical optimization but face greater resistance when recommendations alter antimicrobial spectrum or duration. |
| Evidence use and strategic framing of recommendations | Presenting culture and sensitivity results; using local antibiogram data; framing recommendation around creatinine rise or patient safety; saying "consider narrowing" rather than "this antibiotic is unnecessary"; timing recommendation during rounds; aligning with ID specialist when available. | Evidence-based justification; non-confrontational communication; recommendation framed as shared patient-safety decision; interdisciplinary alignment; timing within live decision-making moments. | Credibility activation; threat reduction; shared decision-making; legitimacy amplification; increased receptiveness when evidence is concrete and publicly discussed during rounds. | Cross-cutting mechanism: strategic framing and evidentiary legitimacy strengthen acceptance across contexts. Reported outcome pattern: de-escalation becomes more feasible when supported by microbiological evidence, antibiogram data, ID alignment, or patient-safety framing. |

| Analytical domain | Examples of initial open codes | Interpretive categories | Inferred mechanisms | Final CMO configuration / reported stewardship outcome pattern |
|---|---|---|---|---|
| Workload, staffing constraints, and temporal positioning | No protected time for stewardship; pharmacist expected to cover medication reconciliation, discharge counselling, drug information, and administrative tasks; retrospective chart review; cross-ward coverage; intervention after 48–72 hours rather than during initial prescribing; limited ward presence. | Role dilution; insufficient clinical pharmacy workforce; reduced proactive involvement; temporal displacement from the point of prescribing; stewardship shifted from real-time negotiation to retrospective follow-up. | Reduced opportunity for influence; delayed visibility; diminished confidence to intervene after decisions are made; lower continuity of stewardship review. | Cross-cutting contextual constraint: workload and delayed workflow positioning weaken pharmacist influence. Reported outcome pattern: reduced opportunity to shape initial antimicrobial choice, delayed review, and lower consistency of de-escalation or duration control. |

## Table S3.2. Development and refinement of the programme theory

| Analytic stage | Purpose | Output |
|---|---|---|
| Initial programme theory | To guide early data collection and analysis by proposing that pharmacists' antimicrobial stewardship influence is activated when institutional and workflow conditions legitimize and embed their input. | Initial proposition: clinical expertise alone is insufficient; stewardship authority depends on governance, workflow integration, documentation visibility, and interprofessional receptiveness. |
| Open coding | To remain close to participants' accounts and identify recurring experiences, incidents, and examples of accepted or rejected recommendations. | Open codes related to leadership support, ward embedding, consultant dominance, documentation, risk aversion, dose optimization, de-escalation, discontinuation, workload, and timing of recommendations. |
| Interpretive categorization | To group open codes into broader explanatory categories relevant to antimicrobial decision-making and pharmacist influence. | Categories such as institutional mandate, workflow embedding, professional hierarchy, defensive prescribing, role-congruent interventions, strategic framing, and role dilution. |
| Retroductive reasoning | To infer the mechanisms most likely to explain why similar pharmacist recommendations were accepted in some contexts but resisted in others. | Mechanisms including perceived legitimacy, confidence, accumulated credibility, self-limitation, strategic deference, risk aversion, threat reduction, and legitimacy amplification. |
| Cross-case comparison | To compare patterns across hospital sectors, clinical settings, documentation systems, and levels of stewardship maturity. | Refined CMO configurations explaining why pharmacist influence varied across contexts despite broadly similar clinical expertise. |
| Final CMO configurations | To present the explanatory model generated from the analysis. | Three final configurations: institutional support enabling routine antimicrobial optimization; consultant dominance constraining pharmacists to advisory roles; and clinical uncertainty sustaining broad-spectrum antimicrobial use. |
